# Associations between body mass index, high-sensitivity C-reactive protein, and depressive symptoms: NHANES 2015-2016

**DOI:** 10.3389/fpsyt.2024.1506726

**Published:** 2025-01-13

**Authors:** Yan Zhang, Fengya Zhen, Yaxing Zhang, Cuixia An

**Affiliations:** Department of Psychiatry, The First Hospital of Hebei Medical University, The Mental Health Center of Hebei Medical University, The Mental Health Institute of Hebei Medical University, Shijiazhuang, Hebei, China

**Keywords:** BMI, PHQ-9, HSCRP, DP, inflammation

## Abstract

**Objective:**

Studies have shown associations between Body Mass Index (BMI), High-Sensitivity C-reactive protein (HSCRP), and depressive symptoms(DP). However, the complex relationship between them remains uncertain. The objective of this research is to examine the correlation between them in a substantial sample that is representative of the national level.

**Methods:**

Our analysis was based on the 2015-2016National Health and Nutrition Examination Survey (NHANES).DP was measured by the Patient Health Questionnaire-9 (PHQ-9). Using multivariable logistic regression analysis and stratified analysis, we examined the relationship between BMI, HSCRP, and DP. We applied generalized additive models to explore the non-linear relationships among variables.

**Results:**

This study included a total of 4834 participants. The results revealed that BMI (*P*=0.002) and HSCRP (*P*=0.008) were risk factors for DP. The relationship between BMI and DP (*P*=0.035), BMI and HSCRP (*P*<0.001) were non-linear. The nonlinear association between HSCRP and DP (*P*=0.031), BMI and DP (*P*=9e-04) is significant in females when stratified by gender. No nonlinear association was found between BMI and DP (*P* =0.677) and between HSCRP and DP (*P* =0.439) in males. The results of the interaction test reveal a significant interaction between HSCRP and gender.

**Conclusions:**

Research has found both BMI and HSCRP are risk factors for DP and the relationship between them was non-linear. The nonlinear associations between BMI and DP, as well as between HSCRP and DP, are gender-dependent.

## Introduction

Depression is a mental disorder that causes functional disabilities and a lack of interest in daily activities, affecting more than 260 million people around the world (1, 2). It is widely known that several factors contribute to depression’s etiology, with obesity playing a substantial role as a contributing factor. The number of obese individuals worldwide exceeds 650 million according to current data, as reported by the World Health Organization. Studies suggest that obesity is linked to depressive symptoms (DP) (3–5). BMI, as a commonly used indicator for assessing the level of obesity in individuals, has been widely employed in research examining the impact of obesity on health (6). Additionally, a study found that adults with high HSCRP were more likely to have depressive symptoms compared to those with low HSCRP (7).

The pathogenesis of DP may also be influenced by inflammation. Inflammation leads to the release of C-reactive protein (CRP) by the liver. Depression and CRP may be linked (8). High-sensitivity CRP (HSCRP) is a more precise biomarker for detecting low-grade systemic inflammation compared to C-reactive protein. The American Heart Association recognizes a threshold of 3 mg/L for low-grade inflammation (9). Research has demonstrated that persistent low-level peripheral inflammation can trigger sustained neuroinflammatory responses, potentially leading to changes in brain structure and function (10). Additionally, Chronic low-grade inflammation has also been proposed to contribute to the altered pathophysiology observed in depression (11, 12). A cohort study shows that serum HSCRP is an independent risk marker for new-onset major depressive disorder in women (13). Another study has found that around one-third of patients who have been diagnosed with depression exhibit HSCRP that is above a specific threshold (14). However, the available clinical evidence regarding these remains is not sufficient. The current research falls short in exploring gender differences. For instance, one study included only a female population and found that HSCRP is an independent prognostic marker for severe depression in women (13); another study did not fully utilize CRP as a continuous variable to deeply analyze its relationship with depression (14). The measurement of HSCRP may facilitate the identification of individuals at heightened risk for the development of depression, offering a rationale for early intervention. In addition, exploring the relationship between HSCRP and depression may provide a scientific basis for the use of anti-inflammatory drugs in depressed patients.

There are also associations between obesity and inflammation (15–19). In detail, these findings indicate that overweight markedly elevates the risk of clinically significant increases in HSCRP, particularly pronounced in individuals with obesity (BMI ≥30 kg/m²). The association between them varies across gender, ethnicity, and age. For instance, the Pearson correlation coefficients between BMI and the logarithmically transformed CRP are 0.360 and 0.370 for adults and children, respectively, with a 0.24 higher correlation for females than males, and a 0.15 higher correlation for North American/European individuals compared to Asians. Moreover, in individuals with obesity, HSCRP is significantly increased and elevated IL-6 is observed across all obesity categories. In summary, both BMI and HSCRP are associated with depression. Given the close relationship between obesity and inflammation, we hypothesized that BMI might mediate the association between HSCRP and DP. Therefore, we conducted a cross-sectional study using data from one NHANES survey cycle between 2015 and 2016. The objective of the research is to analyze the correlation between BMI, HSCRP, and DP and evaluate their impact across diverse populations.

## Methods

### Study population

**Figure 1:**
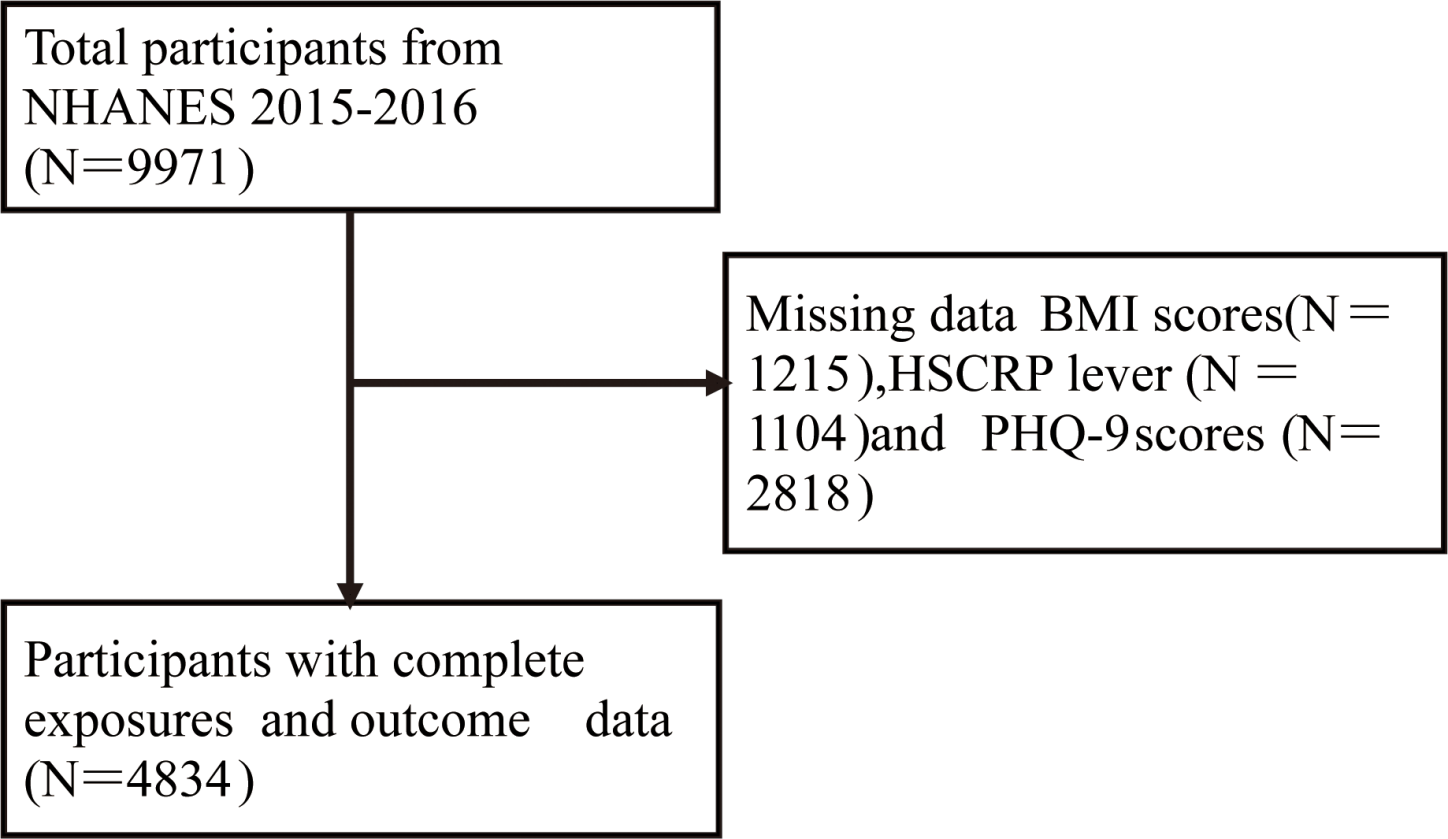
A flowchart of study population selection. NHANES, National Health and Nutrition Examination Survey; BMI, Body Mass Index; HSCRP, High-Sensitivity C-Reactive Protein; PHQ-9, Patient Health Questionnaire-9.

Datasets were obtained from the NHANES, a survey that uses a probabilistic sampling design to ensure a representative sample. Research ethics review board approval was obtained and all participants provided informed consent. One survey cycle covering the period between 2015 and 2016 was analyzed. 9971 participants were initially enrolled in the cohort. After excluding 1215 participants due to missing BMI data, 1104 for the absence of HSCRP data, and 2818 individuals lacking PHQ-9 data, a total of 4834 subjects were included in the analysis, as depicted in **Figure 1**.

### Assessment of DP

PHQ-9 is a validated tool for screening DP. The PHQ-9 score ranges from 0 to 27, with higher scores indicating more severe depressive symptoms (20). The PHQ-9 categorizes depression severity into four categories: mild (score of 5), moderate (score of 10), moderately severe (score of 15), and severe (score of 20) (21), each item on the PHQ-9 is scored from 0 (“not at all”) to 3 (“nearly every day”), assessing the frequency of depressive symptoms experienced during the last two weeks (22). Responses of “Refused” or “Do not know” were treated as missing data. If the PHQ-9 score is ≥10, then the subject is classified as having depression (21). The sensitivity and specificity of a PHQ-9 score ≥10 for severe depression are 88% and 88%, respectively (21). Participants were classified into depressed and non-depressed groups based on PHQ-9 scores.

### Assessment of BMI and HSCRP

BMI measurements were conducted by trained personnel. HSCRP was determined using a near-infrared particle immunoassay rate method.

### Assessment of covariates

We adopted the following criteria for selecting confounders: (a)These variables have previously been shown to be important factors in the association between BMI, HSCRP, and depression (23). Age was included based on this criterion; (b) These confounders were based on a change in the effect estimate exceeding 10% (24). Following this criterion, diabetes, HSCRP, gender, race, sleep disorders, and smoking were chosen as covariates. The physical activity questionnaire (PAQ), derived from the Global Physical Activity Questionnaire and administered at the Mobile Examination Center, was used to assess physical activity levels. These levels were defined by the WHO guidelines on PA (25): inactive (no engagement in strenuous or moderate intensity work activities), moderate (participation in sports without meeting the criteria for strenuous activity), and vigorous (engagement in ≥150 minutes moderate activity per week, ≥75 minutes vigorous exercise per week or strenuous work activities). Alcohol consumption was classified into non-drinkers (complete abstinence from alcohol), moderate drinkers (consumption of up to 2 alcoholic beverages per day for males and up to 1 alcoholic beverage per day for females), and heavy drinkers (consumption of more than 2 alcoholic beverages per day for males and more than 1 alcoholic beverage per day for females). Diabetes was categorized as no diabetes (not having been informed by a doctor of having diabetes) and having diabetes (having been informed by a doctor of having diabetes). Sleep disorders were categorized as having been told by a doctor of having trouble sleeping and not having been told of such difficulties. As for smoking, non-smokers were defined as individuals who have not consumed at least 100 cigarettes in their lifetime, whereas smokers (former smokers and current smokers) were those who have consumed at least 100 cigarettes throughout their lifetime.

### Statistical analysis

The demographic characteristics of participants were analyzed by chi-square tests and the Kruskal-Wallis rank sum test for the non-DP subgroup and DP subgroup. For continuous variables, while for count variables with theoretical counts less than 10, Fisher’s exact probability test is applied. The association of DP, BMI, and HSCRP is based on multivariable logistic regression analysis. These models have DP as the dependent variable and BMI and HSCRP as independent variables. We used three models: Model 1 did not adjust for any covariates; Model 2 included sex, age, and race; Model 3 adjusted for gender, age, race, diabetes, sleep disorders and smoking. In addition, we performed gender-stratified analyses to investigate the sex-discrete relationships between DP, BMI, and HSCRP. Separate multivariable logistic regression analyses were employed for men and women, designating DP as the outcome variable, with BMI and HSCRP as predictors, while controlling for the previously discussed confounders. A generalized additive model was employed to examine the non-linear associations between BMI, HSCRP and DP. The model was adjusted for gender, age, race, diabetes, sleep disorders, and smoking to control for potential confounders. When analyzing the associations between BMI, HSCRP, and DP individually, DP, BMI, and HSCRP were included as additional covariates. The impact of BMI and HSCRP thresholds on DP was calculated separately using a two-segment linear regression model based on a smoothed curve. Interaction and stratified analyses were conducted according to five models (Crude: No factors have been adjusted; Model I: race, age; Model I*: race, age, and the interaction terms for the following variables: race; Model II: BMI, race, age, diabetes, sleep disorders and smoking; Model II*: BMI, race, age, diabetes, sleep disorders and smoking and the interaction terms for the following variables: BMI, race). Subgroup examinations were conducted with gender, smoking, hypertension, and sleep disorders as the stratification variables. We did mediation analysis using the product of coefficients method, and calculating the indirect effect of HSCRP on DP through BMI compared with the total effect of HSCRP on DP. The statistical analysis was carried out using R (version 4.2.0) and EmpowerStats (www.empowerstats.net, X&Y solutions, Inc. Boston, Massachusetts). Statistical significance was defined as less than 0.05 on a two-tailed basis.

## Results

### Participant characteristics

**Table 1** shows the characteristics of the baseline participants stratified by depressive symptoms. The average age was 48.400 ± 18.372 years, with males constituting 48.99% of the cohort. The subjects were categorized as follows: non-DP (PHQ-9 scores <10) and DP (PHQ-9 scores≥10) groups. Significant differences were observed in BMI and HSCRP between the two groups(BMI: [29.392 ± 6.986] vs. [31.160 ± 8.468], *p*<0.001; HSCRP: [3.942 ± 6.916] vs. [6.053 ± 12.818], *p <*0.001).

**Table 1:** Characteristics of participants with Non-DP and DP.

Characteristic	Non-DP (n=4425)	DP (n=409)	*P*-value
BMI, kg/m2, mean ± SD	29.392 ± 6.986	31.160 ± 8.468	<0.001
HSCRP, mg/L, mean ± SD	3.942 ± 6.916	6.053 ± 12.818	<0.001
Gender			<0.001
Male, n (%)	(2202) 49.763	(166) 40.587	
Female	(2223) 50.237	(243) 59.413	
Age, y, mean ± SD	48.298 ± 18.408	49.504 ± 17.970	0.191
Race/ethnicity, (n) %			0.491
White	(1481) 33.469	(151) 36.919	
Black	(908) 20.520	(82) 20.049	
Mexican American	(821) 18.554	(67) 16.381	
Other Race	(1215) 27.458	(109) 26.650	
Educational level, n (%)			<0.001
Less than 9th grade (Includes 12th grade with no diploma)	(1000) 22.599	(135) 33.007	
Some college or AA degree/College graduate or above/More than high school	(976) 22.056	(98) 23.961	
High school graduate/GED or equivalent	(2448) 55.322	(176) 43.032	
Missing data	(1) 0.023	(0) 0.000	
Marital status, n (%)			<0.001
Married	(2209) 49.921	(136) 33.252	
Widowed	(287) 6.486	(32) 7.824	
Divorced	(442) 9.989	(60) 14.670	
Separated	(136) 3.073	(29) 7.090	
Never married	(741) 16.746	(97) 23.716	
Living with partner	(405) 9.153	(40) 9.780	
Missing data	(205) 4.633	(15) 3.667	
Physical activity, n (%)			0.158
Inactive	(2494) 56.362	(235) 57.457	
Moderate	(345) 7.797	(37) 9.046	
Vigorous	(1570) 35.480	(133) 32.518	
Missing data	(16) 0.362	(4) 0.978	
Alcohol consumption			<0.001
Non-drinker	(827) 18.689	(58) 14.181	
Moderate alcohol consumption	(1417) 32.023	(100) 24.450	
Heavy alcohol consumption	(1527) 34.508	(176) 43.032	
Missing data	(654) 14.780	(75) 18.337	
HBP			0.543
No hypertension	(2867) 64.791	(254) 62.103	
hypertension	(1512) 34.169	(150) 36.675	
Missing data	(46) 1.040	(5) 1.222	
Diabetes			<0.001
No	(3805) 85.989	(325) 79.462	
Yes	(620) 14.011	(84) 20.538	
Sleep disorders			<0.001
No sleep disorders	(3348) 75.661	(153) 37.408	
sleep disorders	(1075) 24.294	(256) 62.592	
Missing data	(2) 0.045	(0) 0.000	
Smoking			<0.001
Non-smoker	(2650) 59.887	(162) 39.609	
Former smoker	(1022) 23.096	(89) 21.760	
Current smoker	(746) 16.859	(157) 38.386	
Missing data	(7) 0.158	(1) 0.244	

BMI, Body Mass Index; HSCRP, High-sensitivity C-reactive protein.

Continuous variables: For continuous variables, the Kruskal-Wallis rank sum test is used, while for count variables with theoretical counts less than 10, Fisher’s exact probability test is applied.

Variables with categorical data: (N-observe) percentage, Chi-square test provided the P-value.

### Relationships between BMI and HSCRP with DP

According to **Table 2**, An increment of 1Kg/m^2^ in BMI was associated with a respective increase in the risk of DP of 3.2%, 3.0%, and 2.2% across three models considered (Model 1: OR = 1.032, 95% CI: 1.019-1.045, *P*=<1e−5; Model 2: OR = 1.030, 95% CI: 1.017-1.044, *P* <1e−5; Model 3: OR = 1.022, 95% CI: 1.008-1.037, *P* =0.002); Similarly, An increment of 1mg/L in HSCRP was found to be associated with a respective elevation in the risk of developing DP by 2.3%, 2.2%, and 1.4% across three models (Model 1: OR=1.023, 95% CI: 1.013-1.033, *P*<1e−5; Model 2: OR=1.022, 95% CI: 1.012-1.032, *P*=2e−5; Model 3: OR=1.014, 95% CI: 1.004-1.024, *P*=0.008). These results suggest that BMI and HSCRP are risk factors for DP. In our gender-stratified analysis, it was revealed that both BMI (OR=1.036, 95% CI: 1.019-1.054, *P*=4e-5) and HSCRP (OR=1.027, 95% CI: 1.012-1.041, *P*=2.7e-4) are significant risk factors for the onset of DP exclusively in the female population in model3, the detailed information was provided in **Supplementary Tables 1**, **2**.

**Table 2:** The association between BMI and HSCRP with the risk of DP using multivariable logistic regression.

Variable	Model 1 [OR (95% CI)]	Model 2 [OR (95% CI)]	Model 3 [OR (95% CI)]
BMI(Kg/m^2^)	1.032 (1.019, 1.045)	1.030 (1.017, 1.044)	1.022 (1.008, 1.037)
HSCRP (mg/L)	1.023 (1.013, 1.033)	1.022 (1.012, 1.032)	1.014 (1.004, 1.024)

Model 1: did not adjust for any covariates; Model 2: adjustments were made for sex, age, and race; Model 3: gender, age, race, diabetes, sleep disorders and smoking were adjusted. Significant differences were indicated by a *P* value of 0.05. OR Odds ratio,95% CI, 95% confidence interval.

### Nonlinear associations between BMI, HSCRP, and DP

**Figure 2:**
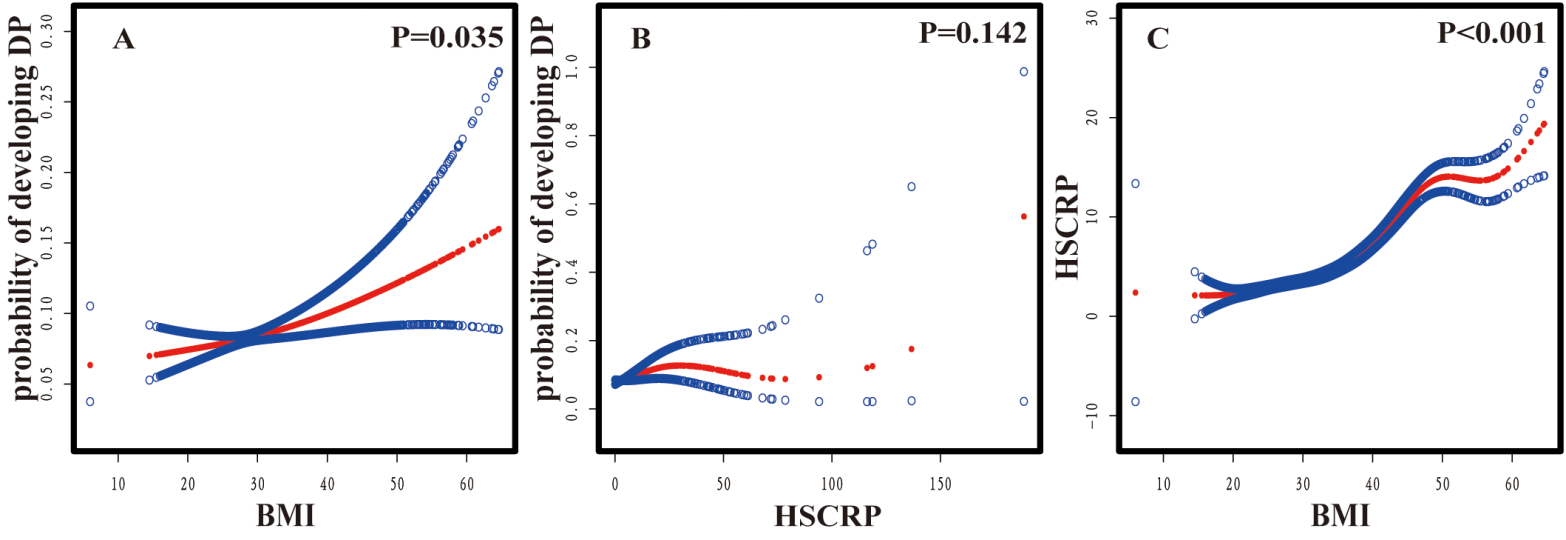
Nonlinear associations between BMI, HSCRP, and DP. **(A)** BMI and probability of developing DP; **(B)** HSCRP and probability of developing DP; **(C)** BMI and HSCRP.The vertical scale ranges from 0.0 (no DP) to 1.0 (DP occurrence). Red lines indicate a smooth curve fit between variables, and blue bands indicate 95%CI. Gender, age, race, diabetes, sleep disorders, and smoking were adjusted. Adjusted model+HSCRP **(A)**, Adjusted model+BMI **(B)**, and Adjusted model+DP **(C)** were each included as confounders.

**Figure 3:**
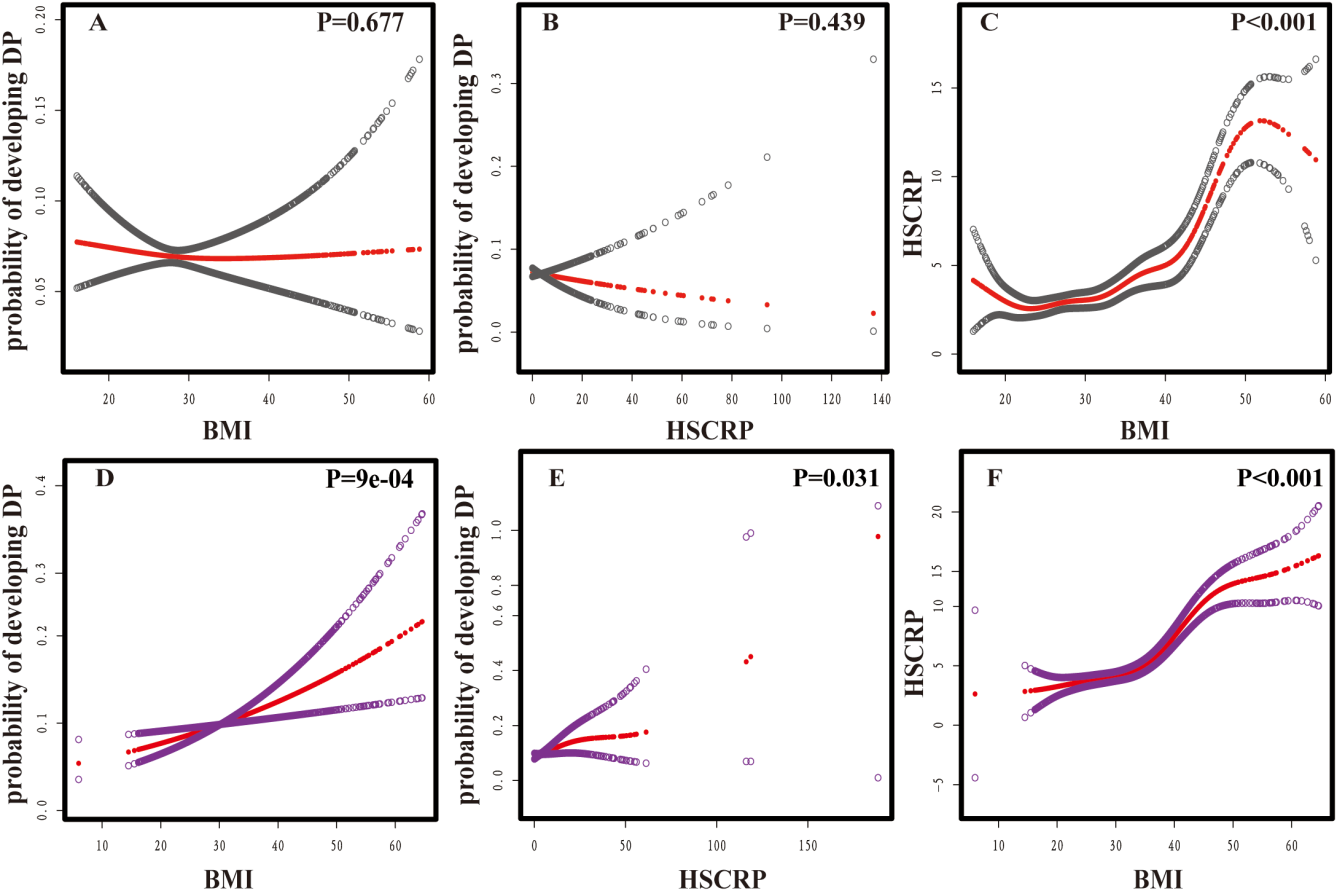
Nonlinear associations between BMI, HSCRP, and DP. **(A)** BMI and probability of developing DP in males; **(B)** HSCRP and probability of developing DP in males; **(C)** BMI and HSCRP in males. **(D)** BMI and probability of developing DP in females; **(E)** HSCRP and probability of developing DP in females; **(F)** BMI and HSCRP in females. The vertical scale ranges from 0.0 (no DP) to 1.0 (DP occurrence). Red lines indicate a smooth curve fit between variables; Grey bands indicate 95%CI in males; Purple bands indicate 95%CI in males. Gender, age, race, diabetes, sleep disorders, and smoking were adjusted. In the analyses of these non-linear associations, HSCRP **(A, D)**, BMI **(B, E)**, and DP **(C, F)** were each included as additional covariates.

Smooth curve fitting analyses provided insight into the nonlinear relationships between BMI, HSCRP, and DP (**Figure 2**). A consistent of confounders were adjusted for all analyses: sex, age, race, diabetes, sleep disorders, and smoking. In detail, the relationship between BMI and DP (*P*=0.035) was further modified for HSCRP; the correlation between HSCRP and DP (*P* =0.142) was additionally adjusted for BMI; and the association between BMI and HSCRP (*P*<0.001) was further refined by adjusting for DP (26, 27). After stratifying by gender there was no significant association between HSCRP and DP in males (*P*=0.439) as well as between BMI and DP in males (*P*=0.677). However, the nonlinear association between HSCRP and DP(*P*=0.031), BMI, and DP (*P*=9e-04) was significant in females (**Figure 3**).

### Threshold effect analysis

In instances where the BMI was below 27.7, this effect is not statistically significant (*P* = 0.327). When BMI exceeded 27.7, the effect coefficient was 1.030, indicating that within this stratum, increments in BMI correlate with an elevated risk of DP, and this association was statistically significant (*P* = 0.002). The log-likelihood ratio test yields a *P*-value of 0.068; About HSCRP and DP, below 14.4 this threshold, the effect of HSCRP on DP was statistically significant, with an OR of 1.034 (95% CI: 1.004, 1.065; *P* = 0.028), above the threshold, the influence of HSCRP on DP was not statistically significant, with an OR of 1.003 (95% CI: 0.989, 1.017; *P* = 0.659). However, the difference in effect between the two segments (below and above the threshold) was not statistically significant, with an OR of 0.970 (95% CI: 0.935, 1.006; *P* = 0.106), therefore, the evidence for a significant change in the relationship between HSCRP and DP at this threshold was weak, as the difference in effects was not significant and the model fit is not improved. Further details are provided in the **Supplementary Table 3**.

### Interaction and stratified analyses

**Table 3** revealed that gender played an interactive role in the association between HSCRP and DP. The female had higher ORs between HSCRP and DP (OR =1.019; 95%CI, 1.004-1.034; *P* =0.011) than the male (OR =0.992; 95% CI, 0.970-1.014;*P* =0.487) in Model II*. In the other four models, similar results were obtained. In detail, among females, across all models, the OR for females was all greater than 1, with the 95% CI not encompassing 1, indicating that the association between heightened HSCRP and an elevated risk of DP is statistically significant; Among males, although a trend towards a decreased risk of depression with elevated HSCRP was observed, this observation did not achieve statistical significance. For model II and model II*, the OR for males was slightly below 1, with the 95% CI including 1, signifying that the association is not statistically significant. Similarly, we analyzed the interaction between gender and BMI to explore whether this interaction impacts the likelihood of DP. We found that in females, there was a positive correlation between BMI and the risk of DP (OR =1.026; 95% CI, 1.008, 1.044; *P*=0.005).In males, although there was a trend towards a reduced risk of DP with increasing BMI, this observation did not achieve statistical significance. (*P* interaction =0.810)in Model II* (**Supplementary Table 4**). In addition, we incorporated factors known to be associated with depression from the existing literature, such as hypertension, smoking, age, and sleep disorders (28, 29) to conduct analyses. The findings suggested a potential interaction between BMI and hypertension, and although its influence on DP was not statistically significant; No significant interaction was observed between smoking and BMI; Age might slightly modify the relationship between BMI and DP in some models, though the significance of this interaction was weak; Sleep disorders didn’t show a significant interaction with BMI about DP across most models; As for HSCRP and DP, no significant interaction between hypertension and HSCRP across all examined models; Upon adjustment for BMI, sex, age, and race, a significant positive correlation emerged between former smoking and DP. Furthermore, the inclusion of interaction terms for BMI, sex, and race indicated a significant moderating effect of smoking on the relationship between HSCRP and DP, which was also significant with the consideration of diabetes, sleep disorders, and other potential confounders and interaction terms; There was no significant difference between HSCRP and DP in different age groups; In the analysis stratified by the presence or absence of sleep disorders, no significant difference was detected in the relationship between HSCRP and DP between the two groups. Further details are provided in the **Supplementary Tables 4**-**12**.

**Table 3:** Interaction between HSCRP and gender on DP in the NHANES 2015–2016.

Gender	Crude[OR (95% CI)]*P*-value	Model I [OR (95% CI)] *P*-value	Model I* [OR (95% CI)] *P*-value	Model II [OR (95% CI)] *P*-value	Model II*[OR (95% CI)] *P*-value
Male	1.006(0.986, 1.026) 0.539	1.006 (0.986, 1.026) 0.568	1.005 (0.985, 1.026) 0.618	0.991 (0.968, 1.014) 0.417	0.992 (0.970, 1.014) 0.487
Female	1.031 (1.017, 1.045) <1e-4	1.031 (1.018, 1.046) <1e-4	1.033 (1.019, 1.047) <1e-4	1.020 (1.006, 1.035) 0.005	1.019 (1.004, 1.034) 0.011
P interaction	0.031	0.026	0.017	0.013	0.031

Crude: No factors have been adjusted;Model I: race, age; Model I*:race, age, and the interaction terms for the following variables: race; Model II: BMI, race,age, diabetes, sleep disorders and smoking; Model II*: BMI, race, age, diabetes, sleep disorders and smoking and the interaction terms for the following variables: BMI, race. Significant differences were indicated by a *P* value of 0.05. OR: Odds ratio,95% CI, 95% confidence interval.

### Mediation of BMI among the correlation between HSCRP and DP

Participants were stratified into two subgroups for analysis: a no-DP group (PHQ-9 < 10) and a DP group (PHQ-9 ≥10). We conducted an analysis to examine the mediating effect of HSCRP through BMI on DP. The results revealed a specific indirect effect of HSCRP through BMI on DP, with a coefficient estimate of OR=1.193e-3, and a 95% CI ranging from 1.160e-4 to 2.407e-3. The *P*-value was 0.032, indicating a statistically significant mediating role of BMI in the relationship between HSCRP and DP. This mediating effect accounted for 28.4% of the total effect(**Supplementary Table 13**). We further conducted separate mediation analyses for different gender subgroups. We found the association between HSCRP and the risk of DP may exhibit gender disparities, with BMI potentially acting as a mediator in females, whereas this mediating role is not apparent in males. Detailed results were presented in the **Supplementary Tables 14**, **15**.

## Discussion

This study investigates the association between BMI, HSCRP, and DP. Multivariable logistic regression analysis provided strong evidence supporting the significant relationship of both BMI and HSCRP with DP. Furthermore, we identified non-linear correlations between BMI, DP, and HSCRP, with gender differences observed in these relationships. Subsequently, we investigated the interaction effect between BMI, HSCRP, and gender on the risk of DP. The results show a significant interaction between HSCRP and gender. Further stratified analysis revealed a significant relationship between HSCRP and DP in females, this may be related to fluctuations in ovarian hormones in women, for example, it has been found that fluctuations in ovarian hormones can regulate women’s susceptibility to stress and inflammation, resulting in DP (30); There is another reason, for example, that women may experience higher levels of stress, which is thought to increase inflammation, which can lead to depression (31). The mediation analysis revealed that HSCRP exerts a significant total effect on DP, with a portion of this effect being mediated through BMI. The mediation effect accounts for approximately 28.4% of the total effect. This is consistent with a previous study (32).

There is a nonlinear relationship between BMI and DP, consistent with results from a study in the Han Chinese population (33). From one perspective, increased BMI may lead to a state of chronic low-grade inflammation, which, by impacting brain function, elevates the risk of depression (34, 35). From another perspective, the intricate interplay between BMI and insulin resistance has been proposed as a potential biological pathway for depression (36, 37). Further analysis indicated the presence of a threshold effect between BMI and the risk of DP, such that when BMI is beyond 27.7, there is a marked increase in risk. Our findings suggest that clinicians can identify groups of patients who may benefit from early intervention by monitoring their BMI. Specifically, for individuals with BMI above a certain threshold, early intervention may be more effective. For example, we found that the risk of DP increased significantly when BMI exceeded 27.7. This finding has prompted clinicians to take more proactive preventive measures when these indicators exceed defined thresholds; The study also revealed that the impact of HSCRP on DP is significantly below a threshold of 14.4, but it becomes nonsignificant beyond this point. Despite indications of a threshold effect, the difference in the magnitude of the effect before and after the threshold was not pronounced, and it didn’t significantly enhance the model’s fit. Further research with larger sample sizes may be required to validate these findings.

Furthermore, our study also identified a nonlinear association between BMI and HSCRP. Although some studies did not explicitly explore this non-linear relationship, they did note a correlation between BMI and HSCRP (38–40), to some extent, this supports our conclusion. However, unhealthy eating patterns can lead to increased BMI and inflammation, which in turn are risk factors for depression. Instead, a healthy eating pattern can combat these risk factors, thereby reducing the risk of depression (41). Therefore, adopting a healthy eating pattern as a prevention strategy may not only help with weight loss but may also reduce the incidence of depression.

We are unique in our approach in that we explored the nonlinear association between BMI, HSCRP and DP. Fewer studies have explored the nonlinear nature of this relationship. In addition, our study revealed gender differences in the relationship between BMI and DP, and between HSCRP and DP. This may be related to the following factors, specifically, on one hand, females showed higher levels of inflammation (42) and obesity prevalence (43, 44); On the other hand, depressed women have higher leptin levels, which may be a key factor in the sex-specific association between BMI and DP (45); Similarly, the relationship between HSCRP and DP showed a gender difference. A possible explanation for this difference has to do with the influence of sex hormones, particularly periodic changes in ovarian hormones, which are known to regulate C-reactive protein levels, which in turn affect the state of depression (46, 47). However, this does not mean that inflammation is not a potential underlying factor for depression in males (48).

Nonetheless, this research does exhibit certain limitations. Firstly, due to NHANES’ use of a complex probability sampling design, which aims to represent the non-institutionalized civilian population of the United States, the homeless, military personnel, and residents in remote areas were not included. Despite some potential biases, after all, these groups constitute a small proportion of the overall population, therefore, their impact on the generalizability of the overall results is limited; Secondly, inflammation is not only associated with depression, but specifically treatment-resistant depression (49–51). The NHANES data includes PHQ-9 scores, which provide a measurement of the severity of DP. However, we must acknowledge that NHANES does not collect detailed information on the number of depressive episodes or the history of antidepressant medication use among participants. Consequently, we cannot directly distinguish between treatment-resistant depression and first-episode depression. Future research should consider these additional variables to gain a more comprehensive understanding of the relationship between depression and inflammation; In addition, the American Heart Association recognizes a threshold of 3mg/l for low-grade inflammation, yet in **Table 1**, the HSCRP in both non-DP (3.9) and DP (6.053) were above 3. This is the other limitation of our study. The high HSCRP in both groups indicates that our sample may not be representative of the general population with lower levels of inflammation. Therefore, our results may pertain more specifically to populations with higher inflammation levels and future research should examine the relationship across a broader range of HSCRP. Finally, it should be noted that this is a cross-sectional study, and future large-scale longitudinal research is needed to explore the causal relationships between them; Some data in the questionnaire, such as physical activity time, may be affected by recall bias, leading to inaccurate results; PHQ-9 is used for screening depression, but it is not the gold standard for diagnosing depression (12).

## Conclusions

In conclusion, our results found that HSCRP and BMI are risk factors for the occurrence of DP. There was a nonlinear relationship between BMI and DP, BMI and HSCRP, respectively. After stratifying by gender, the nonlinear association between BMI and DP, as well as HSCRP and DP, no longer exists in males. Interaction and stratified analyses found that increased HSCRP are merely risk factors for DP in females. A larger prospective study with a larger sample size is needed to investigate the causal relationship between them in the future.

## Data Availability

Publicly available datasets were analyzed in this study. This data can be found here: https://wwwn.cdc.gov/nchs/nhanes/continuousnhanes/default.aspx?BeginYear=2015.
