# Supplementary material for: Associations between body mass index, high-sensitivity C-reactive protein, and depressive symptoms: NHANES 2015-2016
Source: Front Psychiatry. 2025 Jan 13;15:1506726. doi: 10.3389/fpsyt.2024.1506726 (PMC11788894; doi:10.3389/fpsyt.2024.1506726)
Supplement: Supplementary file 1 [file SupplementaryFile1.doc]

**Supplementary Table 1 The association between BMI and HSCRP with the risk of DP using multivariable logistic regression in females.**

| **Variable** | **Model 1 [OR (95% CI)]*P*-value** | **Model 2 [OR (95% CI)]*P*-value** | **Model 3 [OR (95% CI)]*P*-value** |
| --- | --- | --- | --- |
| BMI(Kg/m^2^) | 1.039 (1.023, 1.055) <1e-5 | 1.043 (1.026, 1.059) <1e-5 | 1.036 (1.019, 1.054) 4e-5 |
| HSCRP (mg/L) | 1.031 (1.017, 1.045) 1e-5 | 1.033 (1.019, 1.047) <1e-5 | 1.027 (1.012, 1.041) 2.7e-4 |

Model 1: did not adjust for any covariates; Model 2: adjustments were made for age and race; Model 3: age, race, diabetes, sleep disorders and smoking were adjusted. Significant differences were indicated by a *P* value of 0.05. OR Odds ratio,95% CI, 95% confidence interval.

**Supplementary Table 2 The association between BMI and HSCRP with the risk of DP using multivariable logistic regression in males.**

| **Variable** | **Model 1 [OR (95% CI)]*P*-value** | **Model 2 [OR (95% CI)]*P*-value** | **Model 3 [OR (95% CI)]*P*-value** |
| --- | --- | --- | --- |
| BMI(Kg/m^2^) | 1.009 (0.985, 1.034) 0.478 | 1.008 (0.983, 1.033) 0.550 | 0.995 (0.970, 1.022) 0.731 |
| HSCRP (mg/L) | 1.006 (0.986, 1.026) 0.539 | 1.005 (0.985, 1.025) 0.645 | 0.991 (0.969, 1.013) 0.429 |

Model 1: did not adjust for any covariates; Model 2: adjustments were made for age and race; Model 3: age, race, diabetes, sleep disorders, and smoking were adjusted. Significant differences were indicated by a *P* value of 0.05. OR Odds ratio,95% CI, 95% confidence interval.

**SupplementaryTable 3 Threshold effect analysis of BMI, HSCRP on DP using piece-wise linear regression.**

|  | **BMI^a^＜27.7** | **BMI^a^＞27.7** | **HSCRP^b^＜14.4** | **HSCRP^b^＞14.4** |
| --- | --- | --- | --- | --- |
| **[OR(95%CI)]*P*-value** | 0.978(0.934,1.023) 0.327 | 1.030(1.011,1.050) 0.002 | 1.034 (1.004, 1.065) 0.028 | 1.003 (0.989, 1.017)  0.659 |

a: gender, age, race, diabetes, sleep disorders, smoking and HSCRP were adjusted for all analyses.

b: gender, age, race, diabetes, sleep disorders, smoking and BMI were adjusted for all analyses.

**SupplementaryTable 4** **Interaction between BMI and gender on DP in the NHANES 2015–2016.**

| **Gender** | **Crude[OR (95% CI)]*P*-value** | **Model I [OR (95% CI)] *P*-value** | **Model II [OR (95% CI)] *P*-value** | **Model II*[OR (95% CI)] *P*-value** |
| --- | --- | --- | --- | --- |
| Male | 1.009(0.985,1.034) 0.478 | 1.009 (0.985, 1.034) 0.474 | 0.995 (0.970, 1.020) 0.678 | 0.997 (0.971, 1.023) 0.810 |
| Female | 1.039 (1.023, 1.055) <1e-4 | 1.040 (1.024, 1.057) <1e-4 | 1.030 (1.012, 1.048) 9e-4 | 1.026 (1.008, 1.044) 4.6e-3 |
| P interaction | 0.044 | 0.036 | 0.021 | 0.068 |

Crude: did not adjust for any covariates;

Model I: race, age;

Model II: HSCRP, race,age, diabetes, sleep disorders and smoking;

Model II*: HSCRP, race,age, diabetes, sleep disorders and smoking and the interaction terms for the following variables: HSCRP, age, sleep disorders and smoking;

Significant differences were indicated by a *P* value of 0.05. OR: Odds ratio,95% CI, 95% confidence interval.

**Supplementary Table 5 Interaction between BMI and HBP on DP in the NHANES 2015–2016.**

| **hypertension** | **crude [OR (95% CI)]*P*-value** | **Model 1 [OR (95% CI)]*P*-value** | **Model I* [OR (95%CI)]*P*-value** | **Model II[OR (95% CI)]*P*-value** | **Model II*[OR (95% CI)]*P*-value** |
| --- | --- | --- | --- | --- | --- |
| No hypertension | 1.030(1.012,1.048) 9e-4 | 1.021(1.003,1.040) 0.024 | 1.019 (1.001, 1.038) 0.042 | 1.015 (0.996, 1.034) 0.127 | 1.013 (0.994, 1.032) 0.192 |
| hypertension | 1.034(1.013,1.056) 0.001 | 1.028(1.007,1.050) 0.009 | 1.031 (1.009, 1.054) 0.005 | 1.022 (1.000, 1.045) 0.048 | 1.026 (1.003, 1.050) 0.027 |
| P interaction | 0.745 | 0.613 | 0.406 | 0.603 | 0.385 |

Crude: did not adjust for any covariates;

Model I:HSCRP, race, gender, age;
Model I*:HSCRP,race, gender, age and the interaction terms for the following variables: HSCRP;

Model II: race, gender, diabetes, age, sleep disorders , smoking and HSCRP;

Model II* : HSCRP,age, race, gender, diabetes, sleep disorders and smoking and the interaction terms for the following variables: HSCRP.

**Supplementary Table6 Interaction between BMI and smoking on DP in the NHANES 2015–2016.**

| **smoking** | **crude [OR (95% CI)]*P*-value** | **Model 1 [OR (95% CI)]*P*-value** | **Model I* [OR (95% CI)]*P*-value** | **Model II[OR (95% CI)]*P*-value** | **Model II*[OR (95% CI)]*P*-value** |
| --- | --- | --- | --- | --- | --- |
| Non-smoker | 1.049(1.029,1.069) <1e-4 | 1.043(1.023,1.063) <1e-4 | 1.042(1.021,1.063) <1e-4 | 1.029 (1.009, 1.050) 0.004 | 1.029 (1.008, 1.051) 0.007 |
| Former smoker | 1.026(0.996,1.057) 0.095 | 1.018(0.988,1.049) 0.253 | 1.011(0.979,1.043) 0.516 | 1.006 (0.976, 1.038) 0.686 | 0.998 (0.966, 1.032) 0.910 |
| Current smoker | 1.023 (0.999, 1.048) 0.057 | 1.014 (0.990, 1.038) 0.265 | 1.024 (0.998, 1.050) 0.076 | 1.008 (0.983, 1.033) 0.553 | 1.019 (0.992, 1.046) 0.177 |
| P interaction | 0.362 | 0.254 | 0.400 | 0.460 | 0.482 |

Crude: did not adjust for any covariates;

Model I: HSCRP, race, gender, age;
Model I*:HSCRP, race, gender, age and the interaction terms for the following variables: HSCRP, race, gender, age;

model II: HSCRP, race, gender, diabetes, sleep disorders and age;

Model II*:HSCRP, age, race, gender, diabetes and sleep disorders and the interaction terms for the following variables: HSCRP, age, race, gender, diabetes, sleep disorders.

**Supplementary Table 7 Interaction between BMI and age on DP in the NHANES 2015–2016.**

| **age** | **crude [OR (95% CI)]*P*-value** | **Model 1 [OR (95% CI)]*P*-value** | **Model I* [OR (95% CI)]*P*-value** | **Model II[OR (95% CI)]*P*-value** | **Model II*[OR (95% CI)]*P*-value** |
| --- | --- | --- | --- | --- | --- |
| age.P25: <=P25 | 1.038 (1.023, 1.054) <1e-4 | 1.030 (1.014, 1.047) 2e-4 | 1.032 (1.016, 1.049) <1e-4 | 1.025 (1.008, 1.042) 0.004 | 1.028 (1.011, 1.046) 0.001 |
| age.P25: >P25 | 1.011 (0.984, 1.039) 0.412 | 1.005 (0.978, 1.033) 0.717 | 1.003 (0.976, 1.031) 0.838 | 0.998 (0.970, 1.027) 0.913 | 0.991 (0.960, 1.022) 0.569 |
| *P* interaction | 0.095 | 0.106 | 0.065 | 0.107 | 0.040 |

Crude: did not adjust for any covariates;

Model I :HSCRP, race, gender; 
Model I*:HSCRP, race, gender, age and the interaction terms for the following variables: BMI, race, gender;

Model II: HSCRP, race, gender, diabetes, smoking and sleep disorders;

Model II*:HSCRP, race, gender, diabetes, smoking and sleep disorders and the interaction terms for the following variables: HSCRP, race, gender, diabetes, smoking and sleep disorders.

**Supplementary Table 8 Interaction between BMI and sleep disorders on DP in the NHANES 2015–2016.**

| **Sleep disorders** | **crude [OR (95% CI)]*P*-value** | **Model 1 [OR (95% CI)]*P*-value** | **Model I* [OR (95% CI)]*P*-value** | **Model II[OR (95% CI)]*P*-value** | **Model II*[OR (95% CI)]*P*-value** |
| --- | --- | --- | --- | --- | --- |
| No sleep disorders | 1.018(0.995,1.041) 0.125 | 1.013(0.991,1.037) 0.251 | 1.011 (0.988, 1.035) 0.337 | 1.013 (0.990, 1.037) 0.256 | 1.012 (0.987, 1.037) 0.362 |
| Sleep disorders | 1.020(1.003,1.037) 0.019 | 1.016(0.998,1.033) 0.078 | 1.017 (1.000, 1.035) 0.056 | 1.021 (1.003, 1.039) 0.023 | 1.024 (1.005, 1.043) 0.012 |
| P interaction | 0.990 | 0.989 | 0.697 | 0.874 | 0.455 |

Crude: did not adjust for any covariates;

Model I: HSCRP, race, gender, age;

Model I*:HSCRP, race, gender, age and the interaction terms for the following variables: race;

Model II : race, gender, diabetes, age, smoking and HSCRP;

Model II* : HSCRP,age, race, gender, diabetes and smoking and the interaction terms for the following variables: HSCRP, race, diabetes, and smoking.

**Supplementary Table 9 Interaction between HSCRP and HBP on DP in the NHANES 2015–2016.**

| hypertension | **crude [OR (95% CI)]*P*-value** | **Model 1 [OR (95% CI)]*P*-value** | **Model I* [OR (95% CI)]*P*-value** | **Model II[OR (95% CI)]*P*-value** | **Model II*[OR (95% CI)]*P*-value** |
| --- | --- | --- | --- | --- | --- |
| No hypertension | 1.026(1.013,1.039) <1e-4 | 1.020(1.007,1.033) 0.002 | 1.021(1.008,1.034) 0.001 | 1.014 (1.001, 1.026) 0.036 | 1.015 (1.002, 1.028) 0.027 |
| hypertension | 1.015(0.998,1.032) 0.078 | 1.010(0.991,1.029) 0.307 | 1.008 (0.988, 1.028) 0.445 | 1.002 (0.984, 1.021) 0.802 | 1.002 (0.982, 1.022) 0.860 |
| P interaction | 0.316 | 0.353 | 0.255 | 0.312 | 0.275 |

Crude: did not adjust for any covariates;

Model I: BMI, race, gender, age; 
Model I*:BMI, race, gender, age and the interaction terms for the following variables: BMI;

Model II: BMI, race, gender, diabetes, age, sleep disorders and smoking;

Model II*: BMI, race, gender, diabetes, age and sleep disorders, smoking and the interaction terms for the following variables: BMI, race, diabetes, sleep disorders and smoking.

**Supplementary Table 10 Interaction between HSCRP and smoking on DP in the NHANES 2015–2016.**

| **Smoking** | **crude [OR (95% CI)]*P*-value** | **Model 1 [OR (95% CI)]*P*-value** | **Model I* [OR (95% CI)]*P*-value** | **Model II[OR (95% CI)]*P*-value** | **Model II*[OR (95% CI)]*P*-value** |
| --- | --- | --- | --- | --- | --- |
| Non-smoker | 1.031(1.015,1.048) 2e-4 | 1.022(1.005,1.039) 0.010 | 1.019(1.001,1.037) 0.040 | 1.018 (1.000, 1.035) 0.044 | 1.015 (0.997, 1.033) 0.112 |
| Former smoker | 1.031(1.010,1.053) 0.004 | 1.026(1.004,1.049) 0.019 | 1.030(1.006,1.054) 0.013 | 1.019 (0.999, 1.041) 0.065 | 1.022 (1.000, 1.043) 0.046 |
| Current smoker | 1.003 (0.985, 1.021) 0.773 | 0.994 (0.973, 1.015) 0.567 | 0.995 (0.973, 1.017) 0.659 | 0.994 (0.974, 1.015) 0.573 | 0.994 (0.973, 1.015) 0.573 |
| *P* interaction | 0.019 | 0.027 | 0.016 | 0.120 | 0.044 |

Crude: did not adjust for any covariates;

Model I:BMI, race, gender,age;
Model I*:BMI, race, gender,age and the interaction terms for following variables:BMI, race, gender;

Model II : BMI,race,gender,diabetes, sleep disorders and age;

Model II*:BMI,race,gender,diabetes, smoking and age and the interaction terms for following variables: BMI,race, gender,diabetes, smoking and age.

**Supplementary Table 11 Interaction between HSCRP and age on DP in the NHANES 2015–2016.**

| **age** | **crude [OR (95% CI)]*P*-value** | **Model 1 [OR (95% CI)]*P*-value** | **Model I* [OR (95% CI)]*P*-value** | **Model II[OR (95% CI)]*P*-value** | **Model II*[OR (95% CI)]*P*-value** |
| --- | --- | --- | --- | --- | --- |
| age.P25: <=P25 | 1.022 (1.012, 1.033) <1e-4 | 1.018 (1.007, 1.029) 0.001 | 1.018 (1.007, 1.030) 0.001 | 1.013 (1.003, 1.024) 0.015 | 1.015 (1.004, 1.026) 0.008 |
| age.P25: >P25 | 1.023 (0.994, 1.053) 0.120 | 1.006 (0.974, 1.040) 0.703 | 1.019 (0.985, 1.054) 0.288 | 1.018 (0.987, 1.050) 0.266 | 1.018 (0.986, 1.051) 0.279 |
| *P* interaction | 0.948 | 0.500 | 0.985 | 0.782 | 0.860 |

Crude: did not adjust for any covariates;

Model I: BMI, race, gender;
Model I*:BMI, race, gender, and the interaction terms for the following variables: BMI, race, gender;

Model II: BMI, race, gender, diabetes, sleep disorders and smoking;

Model II*:BMI, race, gender, diabetes, sleep disorders and smoking and the interaction terms for the following variables: race and gender.

**Supplementary Table 12 Interaction between HSCRP and sleep disorders on DP in the NHANES 2015–2016.**

| sleep disorders | **crude [OR (95% CI)]*P*-value** | **Model 1 [OR (95% CI)]*P*-value** | **Model I* [OR (95% CI)]*P*-value** | **Model II[OR (95% CI)]*P*-value** | **Model II*[OR (95% CI)]*P*-value** |
| --- | --- | --- | --- | --- | --- |
| No sleep disorders | 1.017(0.996,1.039) 0.117 | 1.012 (0.988, 1.035) 0.328 | 1.011 (0.987, 1.035) 0.375 | 1.003 (0.979, 1.027) 0.815 | 1.002 (0.977, 1.028) 0.858 |
| sleep disorders | 1.016 (1.004, 1.027) 0.007 | 1.013 (1.002, 1.025) 0.023 | 1.014 (1.003, 1.026) 0.017 | 1.012 (1.000, 1.024) 0.045 | 1.013 (1.001, 1.025) 0.037 |
| *P* interaction | 0.994 | 0.990 | 0.776 | 0.782 | 0.450 |

Crude: did not adjust for any covariates;

Model I : BMI, race, gender, age;
Model I*:BMI, race, gender, age and the interaction terms for the following variables: race;

Model II: BMI, race, gender, diabetes, smoking and age;

Model II*:BMI, race, gender, diabetes, smoking and age and the interaction terms for the following variables: BMI, race, gender, diabetes, smoking.

**SupplementaryTable13 Mediation analysis of the association between HSCRP and the risk of DP mediated by BMI.**

| Exposure | Non-adjusted OR(95%CI)  *P*-value | AdjustIOR(95%CI)  *P*-value | AdjustIIOR(95%CI)  *P-*value |
| --- | --- | --- | --- |
| Direct effect | 5.772e-3( 2.234e-3,8.829e-3)  *P*<1e-4 | 5.491e-3(1.863e-3,8.417e-3) 0.002 | 3.004e-3(-8.06e-4,5.779e-3)0.096 |
| Indirect effect | 2.044e-3(8.09e-4,3.495e-3 )  0.002 | 1.812e-3(6.8e-4,3.236e-3)0.004 | 1.193e-3(1.160e-4,2.407e-3)0.032 |
| Total effect | 7.816e-3(4.631e-3,1.0991e-2)  <1.0e-4 | 7.303e-3(4.141e-3, 1.0432e-2)<1.0e-4 | 4.197e-3 (7.38e-4, 6.951e-3)0.014 |

Non-adjusted model: did not adjust for any covariates; AdjustI model: adjustments were made for sex, age, and race; AdjustII: gender, age, race, diabetes, sleep disorders, and smoking were adjusted. Significant differences were indicated by a *P* value of 0.05. OR Odds ratio,95% CI, 95% confidence interval.

**SupplementaryTable 14 Mediation analysis of the association between HSCRP and the risk of DP mediated by BMI in females.**

| **Exposure** | **Non-adjusted OR(95%CI)P-value** | **AdjustI OR(95%CI)P-value** | **AdjustII OR(95%CI)P-value** |
| --- | --- | --- | --- |
| Direct effect | 9.961e-3(3.886e-3,1.5971e-2) 0.008 | 1.036e-2(4.029e-3,1.6413e-2) 0.008 | 7.333e-3(1.589e-3,1.2926e-2)0.028 |
| Indirect effect | 4.031e-3(1.349e-3,7.529e-3) 0.006 | 4.258e-3 (1.698e-3,7.893e-3) <1e-4 | 3.269e-3 (9.65e-4, 6.491e-3) 0.008 |
| Total effect | 1.399e-2 (8.289e-3,2.0454e-2)  <1e-4 | 1.462e-2(8.989e-3,2.123e-2) <1e-4 | 1.060e-2 (5.223e-3, 1.6602e-2)  <1e-4 |

Non-adjusted mode: did not adjust for any covariates; AdjustI model 2: adjustments were made for age, and race; AdjustII model: age, race, diabetes, sleep disorders and smoking were adjusted. Significant differences were indicated by a *P* value of 0.05. OR Odds ratio,95% CI, 95% confidence interval.

**SupplementaryTable 15 Mediation analysis of the association between HSCRP and the risk of DP mediated by BMI in males.**

| **Exposure** | **Non-adjusted OR(95%CI)P-value** | **AdjustI OR(95%CI)P-value** | **AdjustII OR(95%CI)P-value** |
| --- | --- | --- | --- |
| Direct effect | 1.088e-3(-3.499e-3,4.725e-3) 0.602 | 8.530e-4 (-3.882e-3, 4.597e-3)0.694 | -1.558e-3 (-7.023e-3, 2.374e-3)0.416 |
| Indirect effect | 2.610e-4 (-7.49e-4, 1.307e-3)0.586 | 2.040e-4 (-7.58e-4, 1.218e-3)0.652 | -1.630e-4 (-1.053e-3, 6.52e-4)0.742 |
| Total effect | 1.349e-3 (-3.107e-3, 5.030e-3)0.512 | 1.056e-3 (-3.642e-3, 4.761e-3)0.640 | -1.721e-3(-7.147e-3, 2.206e-3)0.364 |

Non-adjusted model: did not adjust for any covariates; AdjustI model 2: adjustments were made for age and race; AdjustII model 3: age, race, diabetes, sleep disorders, and smoking were adjusted. Significant differences were indicated by a *P* value of 0.05. OR Odds ratio,95% CI, 95% confidence interval.
